# Gait Alterations in the Prediction of Metabolic Syndrome in Patients With Schizophrenia: A Pilot Study With PODOSmart ® Insoles

**DOI:** 10.3389/fpsyt.2022.756600

**Published:** 2022-01-27

**Authors:** Dimitris Efthymiou, Dimitrios X. Zekakos, Evangelia Papatriantafyllou, Efthimis Ziagkas, Alexandros N. Petrelis, Emilia Vassilopoulou

**Affiliations:** ^1^Division of Neurosciences, Department of Psychiatry, School of Medicine, Aristotle University of Thessaloniki, Thessaloniki, Greece; ^2^Digitsole SAS, Nancy, France; ^3^Department of Nutritional Sciences and Dietetics, International Hellenic University, Thessaloniki, Greece; ^4^Laboratory of Motor Behaviour, Aristotle University of Thessaloniki, Thessaloniki, Greece

**Keywords:** antipsychotics, metabolic syndrome, gait analysis, inflammation, gaitline, speed, weight, PODOSmart ®

## Abstract

**Background:**

Second-generation antipsychotics (APs) are associated with metabolic syndrome (MetS), characterized by abnormal pro-inflammatory cytokine production and oxidative stress due to the reduced antioxidant systems, and neurological effects, including mobility impairment. This pilot study investigated relationships between inflammatory-metabolic biomarkers, MetS and gait alterations in patients with psychosis treated with APs.

**Methods:**

Patients with psychosis treated with APs, 20 with MetS (MPS group) and 20 without MetS (PS group) were studied, usinganthropometric data, blood measurements and gait analysis performed with the PODOSmart ® gait analysis device.

**Results and Discussion:**

The MPS group had significantly higher mean body mass index (BMI) and arterial blood pressure (BP) than the PS group. PODOSmart ® gait analysis recorded significant differences between groups in pronation-supination at Heel Off (HO), gaitline HO and gaitline Toe Off (TO). Multifactorial elastic net regression models demonstrated significant association with MetS of inflammatory markers, specific AP2 treatment, gender, age; BMI; BP and smoking (accuracy λ = 0.08), and in relation to gait parameters (accuracy λ = 0.750), the three pronation- supination variables, i.e., at HO, flat foot in (AP2 related) and TO, and propulsion speed. The gait parameters were at the edges of the model, thus indicating a more significant role of these parameters compared to the other clinical variables. Early diagnosis of MetS in patients with schizophrenia via identification of gait alterations can be a screening measure for serious cardiovascular complications related to psychosis and APs, to enable timely dietary intervention that can control the pro-inflammatory state and reduce oxidative stress.

## Introduction

“*Don't walk behind me, I may not lead*.

*Don't walk ahead of me, I may not follow*.

*Walk next to me and be my friend.”*

Albert Camus

Schizophrenia is a chronic psychotic disorder characterized by disorganization of thought and behavior, with possible delusions and/or hallucinations, possible signs of negative symptoms and cognitive deficits (1, 2). Second-generation, or atypical antipsychotics (APs) for the treatment of schizophrenia have offered significant benefits to patients, providing greater effectiveness than traditional APs in treating the negative and emotional symptoms of psychosis, with a lower risk of extrapyramidal side effects (3). Other serious side-effects, however, have been documented for the second generation or atypical APs, which are associated with a reduction in life expectancy of up to 20 years in comparison with the general population (4).

One significant complication is the metabolic syndrome (MetS), which is observed in 40% of patients taking second generation APs, and which carries the risk of cardiovascular disease (CVD) and diabetes mellitus (DM), contributing to a decrease in life expectancy (5). MetS is associated with a low-grade inflammation and oxidative stress, that may be modulated with early proper dietary intervention (5).About 50% of patients with schizophrenia are obese, due to a variety of factors, including reduced mobility, drugs that cause significant weight gain (WG), poor eating habits and difficulty in understanding the meaning of proper nutrition (6). Apart from the APs medication, metabolic complications result from the disease itself. WG, type II DM and insulin resistance were first reported to be associated with schizophrenia by Sir Henry Maudsley in 1879, long before the use of second-generation APs (7).

Among the second-generation APs, olanzapine and clozapine are reported to cause the most severe metabolic complications, while aripiprazole, brexpiprazole, cariprazine, lurasidone, and ziprasidone produce milder effects. The metabolic side-effects are correlated with overweight or obesity at baseline weight, male sex, and non-white ethnicity, but also with the effectiveness of the APs in the treatment of psychosis (8).

In addition, both psychosis and APs are associated with a variety of adverse neurological effects, among which is impairment of mobility (9).Changes in mobility can be observed even before the onset of psychosis, and have been documented in children with a family history of schizophrenia (10). Studies have shown that the main gait deficit in schizophrenic bradykinesia is a disturbed regulation of stride length (11). Treatment with conventional APs exacerbated this deficit, but treatment with atypical APs showed no additional effects on the gait of patients with schizophrenia, and their pace (steps per minute) remained largely unaffected (12). The reasons for the impairment of motion in schizophrenia have not yet been fully elucidated. Studies of motor disorders in patients with schizophrenia indicate that at least part of the reduction in motor function may be due to impairment of internal control mechanisms, interfering with the automatic execution of motor tasks (13).

Traditional antipsychotics are reported to cause drug-induced mobility disorders, including neuroleptic-induced parkinsonism, neuroleptic-induced acute dystonia, neuroleptic-induced dementia, neuroleptic-induced late dyskinesia, neuralgia-induced malignant syndrome, and malignant neoplastic syndrome, all of which affect the gait, resulting in slowness, dragging of the feet and impaired balance. Fine movements are also affected, leading to difficulties in the physical execution of daily activities. With the second-generation APs, these neurological side-effects are significantly less common (14).

Gait consists of a series of rhythmical, alternating movements of the trunk and limbs that result in the forward progression of the center of gravity, and is a reliable indicator of overall functionality. Gait analysis includes accurate measurements of spatiotemporal and kinematic parameters, for which various devices have been developed, including both laboratory based and portable systems (15). Although these devices have gained popularity in scientific research, they also present shortcomings. Specifically, this type of equipment is not usually portable and it can be used only in laboratory-based measurements. In addition, its configuration is capable of capturing only a limited number of steps (16, 17). The high cost of these devices is also a factor to be considered (18, 19).

Portable smart devices have been developed recently that appear to overcome the limitations of the classical gait analysis devices. The PODOSmart ® device, developed by Digitsole SAS, is a low-cost portable system that consists of insoles with completely wireless sensors and integrated internal storage. They can fit into any shoe and can measure spatial, temporal, and kinematic gait parameters in general and specific populations. The device and the validation of the measured and calculated parameters is described in detail in a paper that is currently under review.

An increasing body of research has used analysis of gait to predict metabolic complications, including type II DM and CVD. A recent meta-analysis suggested the use of gait speed as a predictor of CVD onset and mortality (20).The limited research on gait alterations in MetS revealed an association between slower gait and low levels of high-density lipoprotein cholesterol (HDL) and high fasting glucose levels in women (21), but no further exploration was made.

### Aim of the Study

The purpose of the current pilot study was to investigate whether the MetS in patients with psychosis treated with second-generation APs is associated with alterations in gait, the type of APs medication and other inflammatory-metabolic biomarkers.

## Methods

### Participants

Patients diagnosed with schizophrenia, who were being treated with second-generation APs and monitored at the Thessaloniki Psychiatric Hospital (Greece), were recruited from January to April 2021. The inclusion criteria were: (1) age ≥ 18years, (2) a diagnosis of psychosis according to the ICD-10 classification system, and (3) long-term treatment with second-generation APs medication (**≥**5 years). The exclusion criteria were: (1) a clinical history of substance misuse (dual diagnosis patients), (2) current or past use of typical AP medication, (3) pregnancy, (4) intellectual disability, and (5) a diagnosed chronic medical/metabolic condition other than MetS, such as CVD and DM.

The patients were classified in two groups, based on the following criteria:

1. Patients With Psychosis who met at Least 3 of the Criteria for the Diagnosis of MetS (the MPS Group).
2. Patients With Psychosis Without MetS (the PS Group).

Before inclusion in the study, all the participants were informed in detail about the study protocol and they provided their written informed consent. The study was approved by the Research Ethics Committee of the Aristotle University of Thessaloniki (code number 4/26.01.2021) and complied with the International Code of Medical Ethics of the World Medical Association and the Helsinki Declaration.

### Clinical Assessment

Schizophrenia classification (F20) was made according to the ICD-10 classification by the medical team of the Thessaloniki Psychiatric Hospital and cross-checked by the psychiatrist in the study team. The APs medication prescribed to each of the patients in the study was recorded in detail and subdivided into two categories: AP1, related to a higher risk of WG of ≥ 7% from the baseline weight, namely aripiprazole, amisulpride quetiapine XR, paliperidone, and ziprasidone, and AP2, related to a higher risk of WG ≥ 7% from the baseline weight, namely olanzapine, asenapine, clozapine and risperidone (22–24), as we described previously in detail (25).

### Blood Pressure Determination

Arterial blood pressure (BP) was recorded to the nearest 2 mmHg, using a mercury sphygmomanometer with the arm supported at heart level, after the subject had been sitting quietly for 10 min. One trained member of the research team took three separate readings at 1-min intervals. The average of the last two readings was used for analysis.

### Anthropometric Assessment

Anthropometric measurements were made on all participants on the morning of gait analysis, after fasting for at least 8 h, by one trained investigator. Height was measured to the nearest 0.1 cm, using a commercial stadiometer (Leicester Height Measure, Invicta Plastics Ltd, Oadby, UK) with the participants barefoot, their shoulders in a relaxed position, their arms hanging freely and their heads in the Frankfort horizontal plane. The participants were weighed barefoot and in light clothing to the nearest 0.1 kg, using a TANITA RD-545 (“RD-545-Connected smart scale | Tanita Official Store,” n.d.). Body mass index (BMI) was calculated from the current weight and height [weight (kg) by height squared (m2)]. The waist circumference (WC) was measured with a SECA flexible, inextensible measuring tape with an accuracy of 1 mm, on a horizontal plane, after exhalation, at a point equidistant from the lowest floating rib and the upper border of the iliac crest.

### Biochemical/Hematological Assessment

Venous blood samples were collected from all participants, as part of their routine monitoring procedure on a day independent of the gait analysis, after overnight fasting, and analyzed on the hospital premises, using automatic biochemical analyzers, under standard conditions. The white blood cell count (WBC), and concentrations of blood glucose (GL), serum total cholesterol, triglycerides (TG), high density lipoprotein cholesterol (HDL-C), low density lipoprotein cholesterol (LDL-C), high-sensitivity C reactive protein (hsCRP) and B12 were measured by an automatic analyzer (Toshiba TBA 120FR; Toshiba Medical Systems Co., Ltd., Tokyo, Japan).

### Definition of MetS

MetS was defined according to the International Diabetes Federation (IDF) criteria (25), according to which patients need to have at least three of the following: BMI ≥ 30 or WC ≥ 94 cm in men and ≥ 88 cm in women; TG ≥ 150 mg/dl, or receiving drug treatment for hypertriglyceridaemia; HDL <40 mg/dl in men and HDL <50 mg/dl in women, or receiving drug treatment for hyperlipidemia; BP ≥ 130/80 or receiving drug treatment for hypertension; fasting GL ≥ 100 mg/dl, or known DM, or receiving drug treatment for hyperglycaemia (26).

### Gait Analysis

The PODOSmart ® gait analysis device allows measurement of walking and running parameters under real-world conditions. The PODOSmart ® insoles (Digitsole SAS, Nancy, France) consist of a pair of insoles (available in most shoe sizes) connected to a mobile application via a Bluetooth connection box. The PODOSmart ® smart insole is rechargeable via USB and can be used continuously for more than 33 h. Each PODOSmart ® insole has an inertial platform that can record walking or running steps and the placement of each foot in 3D space. The Bluetooth connection box is used to collect data captured by the smart insoles. The data are processed into a clinically usable dataset by artificial intelligence algorithms to extract spatiotemporal, kinematic, and biomarker parameters that are presented in the interface.

PODOsmart insoles offer gait analysis parameters based on both monopedal and bipedal gait data. Monopedal gait parameters include: the angles of the foot during heel strike (HS), heel-off (HO), flat foot in (FFI) and toe off (TO), the stride length in meters (i.e., the distance of foot displacement between two consecutive steps on the same side); stride duration in milliseconds (i.e., the duration of foot displacement between two consecutives steps on the same side); stance time (i.e., the percentage of duration of contact between the foot and the ground during a one stride cycle); swing time as a percentage of swing duration during a one stride cycle; foot progression angle in degrees (i.e., the angle defined between the orientation of the foot and the user's trajectory). Three gait parameters are calculated from the recorded data on both feet (bipedal): cadence, which represents the number of steps per minute; gait speed (km/h), which is the average walking speed of the user; double contact duration (%), that refers to the duration of simultaneous contact between both feet and the ground.

### Procedure

Prior to gait analysis, the subjects removed their shoes and wiped their feet with alcohol. A study team member placed an insole in each shoe, according to the subject's shoe size. The subjects walked for 1 min at their preferred velocity when walking with comfort, on flat ground, straight ahead, and they were then asked to make a U-turn at the half-way point and return to the starting line.

### Statistical Analysis

Statistical analysis included statistical tests, inferential analysis and modeling. The R software (version 4.04) and R studio (version 1.4.1106) were used. For the descriptive analysis, data were shown as mean and standard deviation (mean±SD) or median and interquartile range, as appropriate. Normality was checked using the Shapiro-Wilk test.

Normally distributed data were compared between the MPS and PS groups by *t*-test and non-normally distributed data by the Wilcoxon test. In all cases, the level of significance (a) was set at 0.05. *P*-values ≤ 0.05 were considered statistically significant.

To further investigate the association of MetS in relation to gait analysis parameters, APs medication, demographic, anthropometric, clinical, and biochemical data, the elastic net regression model was used. This is a regularized regression methodology that combines Lasso and Ridge regression (26). Logistic regression model indicates the conditional probabilities through a linear function of the predictors:


$$Pr(G\;=\;1/\text{x})\;=\;\frac{1}{1\;+\;{\text{e}}^{-(\beta_{0}+x^{T}\beta )}}$$

$$Pr(G\;=\;2/\text{x})\;=\;\frac{1}{1\;+\;{\text{e}}^{+(\beta_{0}\;+\;x^{T}\beta )}}=\;1\;-\text{ Pr}(\text{G }=\;1/\text{x})$$

Alternatively, this implies that


$$\log\frac{Pr(G\;=\;1/x)}{Pr(G\;=\;2/x)}\;=\;\beta_{0}+x^{T}\beta$$

In the regularized maximum binomial likelihood, p(*x*_*i*_) = Pr(G = 1/*x*_*i*_) is the probability for the i observation at a specific value for the parameters (β_0_,β). To maximize the penalized log-likelihood


max( β0,β)∈ℝp + 1[1N∑i = 1NI{(gi = 1)∗logp(xi)                             + I(gi = 2)∗log(1−p(xi))}− λPa(β)]

In the final step of the elastic net, the use of coordinate descent for solving the penalized weighted least-squares problem has as an objective function, the penalized negative binomial log-likelihood, and is:


$$\begin{array}{l} \min_{(\beta_{0},\beta )\;\in \;{\mathbb{R}}^{p\;+\;1}}\text{​ }-\;[\frac{1}{N}\;\sum_{i\;=\;1}^{N}y_{i}\;(\beta_{0}\;+\;x_{i}^{T}\beta )\\ \;-\;log(1\;+\;e^{\left(\beta_{0}\;+\;x_{i}^{T}\beta \right)})]\\ \;+\text{ λ}[(1\;-\text{ α})||\beta |{|}_{2}^{2}/2\;+\text{ α}||\beta |{|}_{1}] \end{array}$$

where λ is a parameter that controls shrinkage, 0 is no penalty and ∞ is entirely penalty, and α regulates how much of the ridge vs. lasso, 0 is the ridge, and 1 is the lasso. In our process, we run multiple cross-validations for different values inside this interval [0,1] to define the optimal α for our model. To apply this methodology in R software, we used the glmnet package (version 4.1-1).

## Results

For the purposes of this pilot study, 20 patients with schizophrenia (80% males) were recruited who were taking second-generation APs and who fulfilled the criteria of MetS (the MPS group), and 20 patients with schizophrenia taking second-generation APs, but without MetS (85.7% males) (the PS group). All the study patients had been taking second-generation APs for a mean of 12.9 ± 7.2years: An AP2 was the selected treatment for 12/20 MPS (60%) and 6/20 of PS, while the rest of the patients were receiving an AP1.

Table 1 shows the demographic characteristics, anthropometric measurements, and clinical data of the patients in the MPS and PS groups. The mean age of the participants was similar in the two groups (48.8 ± 10.98 years for MPS vs. 48.05 ± 15.07 years for PS, *p* > 0.05). The MPS patients (those with MetS) had a significantly higher mean body weight (*p* = 0.04) and BMI (*p* < 0.01), mean diastolic arterial pressure (DAP) (*p* = 0.01) and median systolic arterial pressure (SAP) (*p* < 0.01) than the PS patients. Table 2 summarizes the chemical biomarkers in the two groups. The mean serum level of HDL was lower, and the WC and the mean serum levels of TG and fasting GL were higher in the MPS than in the PS group, but the differences were not statistically significant (p>0.05).

**Table 1:** Study participants' demographic and clinical characteristics.

**Characteristic**	**MPS**	**PS**	***p*-value**
*n*	20	21	
Gender	16M/4F	18M/3F	0.780
Age (years)	48.8 (±10.98)	48.05 ± 15.07	0.857
Weight (kg)	88.7 (±15.15)	78.57 ± 15.79	0.043
Height (m)	1.73 (±0.11)	1.72 ± 0.11	0.718
BMI (kg/m^2^)	29.9 (±6.32)	26.58 ± 4.52	0.059
Waist circumference (cm)	102.85 (±14.14)	100.81 ± 14.02	0.645
Systolic arterial pressure (mmHg)	130 (±9.64)	110 ± 10.38	0.004
Diastolic arterial pressure (mmHg)	81.5 (±9.64)	74.71 ± 8.93	0.014
Cigars/day	15.1 (±8.72)	12 ± 9.17	0.216

*BMI, Body Mass Index*.

**Table 2:** Biochemical parameters between MPS and PS.

	**MPS**	**PS**	***p*-value**
HT (%)	43.05 (±3.66)	42.32 ± 4.19	0.563
RBC (M/ml)	4.94 (±0.5)	4.8 ± 0.52	0.389
PLT (K/ml)*	231 (264)	208 (270)	0.285
B12 (pg/ml)*	297 (484)	298 (542)	0.979
WBC (K/ml)*	8.57 (7.14)	7.24 (8.19)	0.103
hsCRP (mg/dl)*	0.29 (1.02)	0.25 (1.12)	0.441
T-Chol (mg/dl)	162.1 (±440.3)	182.33 (±48.54)	0.170
HDL (mg/dl)	39.15 (±11.52)	42.86 (±9.81)	0.273
LDL (mg/dl)	91.3 (±38.01)	114.47 (±39.6)	0.063
TG (mg/dl)*	123.5 (520)	108 (244)	0.230
Glucose (mg/dl)*	101.5 (127)	93 (57)	0.137

HT, hematocrit; RBC, Red Blood Cells; PLT, Platelets; WBC, White blood cells; hsCRP, high sensitive C-reactive protein; T-Chol, Total Cholesterol; HDL, High Density Cholesterol; LDL, Low Density Cholesterol; TG, triglycerides. Values are Mean (±Standard Deviation) or Median (Interquartile Range) in non-normally distributed variables indicated by* (Wilcoxon Test).

Table 3 shows the comparison of parameters measured with PODOSmart ® during free gait, that presented a statistically significant difference between the MPS and the PS group: pronation and supination HO (p = 0.04), in gaitline HO (p = 0.01) and gaitline TO (*p* = 0.03). The comparison of all measured parameters with PODOSmart ® during free gait are provided in Supplementary Table A of the Supplementary Material.

**Table 3:** Gait analysis variables analyzed with PODOSmart ® which presented statistically significant difference (*p* < 0.05) among the MPS and PS.

	**MPS**	**PS**	***p*-value**
Right.pro_sup_HO.avg	0.06 (±0.11)	0 (±0.08)	0.043
Right.gaitline_TO.avg*	0.06 (3)	0 (3)	0.035
Right.gaitline_TO.std*	0.14 (0.54)	0 (0)	0.035
Left.gaitline.HO.std*	0.11 (0.41)	0.19 (0.49)	0.012

Pro, pronation; Sup: supination; HO, Heel off; TO, Toe off; avg: average; std, standard deviation. Values are Mean (±Standard Deviation) (t-test) or Median (Interquartile Range) in non-normally distributed variables indicated by ^*^ (Wilcoxon test).

**Figure 1:**
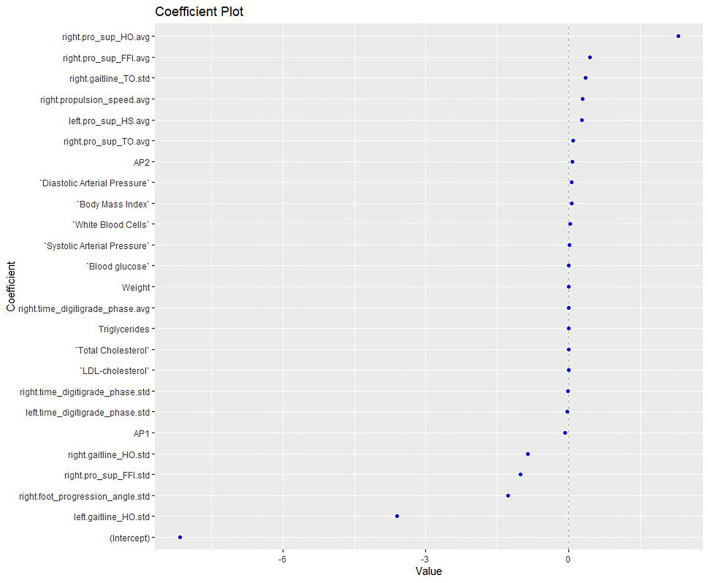
Elastic net model exploring the APs, anthropometric and biomarkers associated with the risk of MetS. HO, Heel off; FFI, Flat foot in; TO, Toe off; HS: heel strike; Pro, pronation; Sup, supination; avg, average; std, standard deviation; AP, antipsychotics; LDL, low-density lipoprotein.

Elastic net regression was used to construct a multifactorial model to explore the APs, anthropometric factors and biomarkers associated with the risk of MetS, as shown in Figure 1. The model presented good accuracy (0.857) and the AUC showed ability of the model to distinguish factors in affecting MetS (AUC: 0.94; 95% CI: 0.74, 1). According to the model, factors associated with a higher risk of MetS in patients with psychosis were: higher levels of hsCRP, GL, TG, and WBC; use of AP2 treatment; female gender; greater age; higher BMI; higher arterial BP; and smoking a greater number of cigarettes. In this case series, AP1 medication, male gender, WC, and high levels of LDL, total cholesterol and B12 were not correlated with MetS.

**Figure 2:**
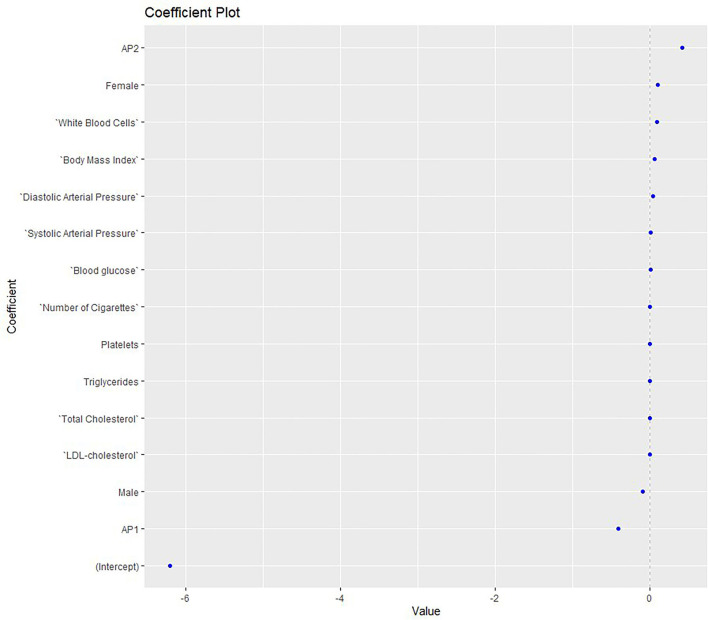
Elastic net model exploring the MetS risk in relation to gait parameters measured with PODOSmart ®, along with anthropometric, clinical and biochemical markers. AP, antipsychotics; LDL, low-density lipoprotein.

A second elastic net model (Figure 2) was constructed to assess the MetS risk in relation to gait parameters measured with PODOSmart ®, along with anthropometric, clinical and biochemical markers. This second model demonstrated accuracy at good levels (0.750), along with an acceptable AUC (AUC: 0.8; 95% CI: 0.6, 1). Of the gait parameters, the two pronation and supination variables (HO and FFI) and gaitline TO, and the propulsion speed, showed a close association with the risk of MetS. Among the clinical and anthropometric factors and biochemical markers, AP2, and higher levels of weight, BMI, systolic and diastolic arterial BP and WBC showed significant positive association with the MetS risk.

Conversely, the MetS risk was reduced when the progression angle, gaitline HO and the pronation-supination FFI increased. AP1 medication was inversely related to MetS, while the levels of LDL and TG showed no effect. The gait parameters were found in the edges of the model with higher positive and negative coefficients compared to the clinical, anthropometric and biochemical biomarkers.

## Discussion

Patients with schizophrenia present various metabolic and inflammatory disturbances related to both the disease itself and the APs medication used (27), leading to an increased risk of MetS (28). Alterations in gait have been studied only rarely, in either schizophrenia (29, 30) or MetS (21), with results suggesting the need for further research. In this, our first pilot study, we aimed to investigate factors in the clinical, metabolic and inflammatory profile of patients with psychosis, through their routine screening, to determine their possible association with gait alteration and MetS and to explore whether gait analysis could be used as an early indicator for metabolic and inflammatory abnormalities in psychosis. We developed two prediction models that detected a close relationship between MetS and known metabolic and inflammatory markers, depending on the APs medication used and lifestyle factors. Alteration in the gait parameters measured with PODOSmart ® showed a strong relationship with MetS in the prediction model; increase in the two parameters of pronation-supination (HO and FFI), gaitline TO, and propulsion speed were positively associated with the MetS. Conversely, increases in gaitline HO, progression angle and the pronation-supination FFI were inversely correlated with MetS.

We observed that patients receiving AP2were at greater risk of developing MetS. A recent meta-analysis reflected the wide variation in the impact of different APs on metabolic dysregulation (8). Clozapine and olanzapine, sub-categorized in the AP2 medication sub-group, have been observed to produce higher rates of WG and greater increase in BMI than AP1 medication, such as aripiprazole and paliperidone. MetS has been documented to contribute to the increased risk of CVD in both the general population (31) and patients with psychosis receiving atypical APs therapy (32, 33); as such the last are suggested to be monitored constantly for metabolic dysregulation in the clinical routine (32). Metabolic dysregulation is evident in drug naive patients with psychosis, indicating that factors other than medication are implicated, including genes (34) and lifestyle choices (35, 36). Studies in recently diagnosed, untreated patients with psychosis indicate that they have up to three times more intra-abdominal fat than the general population. This can be explained by the involvement of the hypothalamic-pituitary-adrenal axis, which regulates, among other functions, the body metabolism and its response to the stress of the development of psychosis (37). Half of the patients have signs of MetS when first diagnosed with psychosis, even before initiation of APs treatment. Medication is also implicated, as, according to Eskelinen and colleagues (2015), the risk of metabolic disorders in clozapine users is doubled (37).The duration of APs treatment, also, is related to an increase in BMI and the occurrence of MetS. In our study population, the prediction model endorses as significant factors for MetS the specific APs treatment, increased BMI, arterial BP, and raised blood levels of GL and TG. Longer duration of psychosis is related to the development of obesity and metabolic symptoms, while according to Dehelean and colleagues (2019) the use of risperidone appears to lead to hypertension and an increase in WC (38).

Although HDL level and WC have been recognized as factors in the diagnosis and prognosis of MetS (39), they did not appear as significant factors in our population. Conversely, two inflammatory markers, hsCRP and WBC, were found to be significantly related with MetS. Patients with psychosis suffer from low-grade chronic inflammation, probably as a stress response of the immune system (40). Various immune and inflammatory alterations have been found, in both brain and blood, among which increases in WBC and hsCRP relevant to metabolic disturbances in psychotic patients (41). A high level of hsCRP during adolescence is a risk factor for psychosis in later life (42). In under-treated patients with psychosis, the type of drug therapy is related to the level of hsCRP and the WBC (43) and under-response to treatment has also been correlated with inflammatory markers (44). As shown by Vassilopoulou et al. (25) the long-term use of AP2 is relevant to increases of hsCRP and WBC. Similarly, Jacomb et al. (45) suggested that hsCRP is elevated in acute psychosis, but also significantly elevates in chronically ill patients with psychosis.

In parallel, schizophrenia and APs both disturb gait parameters in complex ways, as a variety of factors are involved in the alterations. In 1999, Flyckt and colleagues referred to heredity as a factor in the occurrence of neurological abnormalities that gait (44), while other researchers have reported that differences in mobility can be partly explained by the cognitive level of an individual (46). The impact on motor activities and gait performance are often depicted by the speed and balance (47). Analysis of gait using PODOSmart ® uncovered significant differences in gait variables between the two study groups, with and without MetS, including the gaitline at the TO phase and the pronation and supination HO. During the TO phase, pressure on the heel switch is released, activating the stimulator and evoking dorsiflexion of the foot during the swing phase of gait and altering the knee flexion during the swing phase of normal gait (48).

The three pronation and supination variables in PODOSmart ®, namely pronation-supination at heel strike (HO), pronation-supination at flat foot (FFI) and pronation-supination at toe off (TO) represent ankle varus or vagus in the three single limb support phases (initial contact, mid stance, terminal stance). Ankle valgus and varus belong to an insidious deformity that results in pronation or supination of the foot. The causes of varus or valgus ankle vary and may occur due to neuromuscular disorders or skeletal dysplasia (49–53).

The prediction model also correlated propulsion speed with MetS. Propulsion speed as a PODOSmart ® variable refers to the speed of the foot at the TO phase. Propulsion and stance stability, shock absorption and energy conservation all belong to the basic locomotor functions of gait (54).Consequently, regardless of the initial standing phase of the limb, initiation of a step begins with a shifting of body weight and anterior displacement at the ankle joint of the supporting limb. Swinging of the lower limb involves a change in body posture for the propulsion. Hip flexion and ankle dorsiflexion lead to lifting of the swing limb, creating anterior forces that modify the standing balance. Rapid hip flexion offers further acceleration that augments this effect (55, 56).

Motor side-effects significantly affect the autonomy and functionality of the individual in their daily living activities. Psychosis and some types of APs medication have been associated with a variety of mobility side-effects, but also with metabolic and inflammatory dysregulation, all of which can adversely affect the quality of life and morbidity and mortality rates. Gait disturbances can reduce mobility and therefore increase MetS and inflammatory complications (57, 58). In general, the assessment of motor dysfunction and gait disorders using clinical tools is a challenging process (58, 59). We have demonstrated in our model that gait parameters calculated by PODOSmart ® have a stronger association with MetS than clinical, anthropometric and biochemical variables.

A limitation of the current investigation is the small number of participants and the absence of statistically significant differences in the biomarkers determining MetS between the two subgroups of patients taking different second-generation APs. In view of the documented link between psychosis, APs and inflammatory and metabolic disturbances and altered gait patterns, however, it is expected that further investigation with larger samples will provide valid information and better understanding, enhancing the ability to interpret these alterations.

Future investigation of the metabolic and inflammatory parameters related to schizophrenia and APs medication, and their association with impairment of motor-gait parameters may provide information about the pathophysiological complications of schizophrenia and the mechanisms leading to the differences in motor performance. Gait analysis with PODOSmart® is a low-cost method, rapid and simple to use method and replicated in any space available. Early diagnosis of MetS via gait alterations in patients with schizophrenia can be implemented for timely preventive measures against serious cardiovascular complications, primarily adaptation of a healthier antioxidant and anti-inflammatory diet and enhancement on the compliance with guidelines for relevant lifestyle changes in patients with psychosis, such as smoking cessation and increase in physical activity.

## Data Availability Statement

The raw data supporting the conclusions of this article will be made available by the authors, without undue reservation.

## Ethics Statement

The studies involving human participants were reviewed and approved by Research Ethics Committee of the Aristotle University of Thessaloniki. The patients/participants provided their written informed consent to participate in this study.

## Author Contributions

DE and EV designed the project and wrote the original article. DE and EP collected the data. DZ and AP analyzed the data. EV, DZ, EZ, and AP interpreted the data. All authors critically revised and approved the final version of the manuscript.

## Funding

This research was funded by Digitsole SAS, Nancy, France.

## Conflict of Interest

The authors declare that the research was conducted in the absence of any commercial or financial relationships that could be construed as a potential conflict of interest.

## Publisher's Note

All claims expressed in this article are solely those of the authors and do not necessarily represent those of their affiliated organizations, or those of the publisher, the editors and the reviewers. Any product that may be evaluated in this article, or claim that may be made by its manufacturer, is not guaranteed or endorsed by the publisher.
