# Supplementary material for: Gait Alterations in the Prediction of Metabolic Syndrome in Patients With Schizophrenia: A Pilot Study With PODOSmart ® Insoles
Source: Front Psychiatry. 2022 Jan 27;13:756600. doi: 10.3389/fpsyt.2022.756600 (PMC8829465; doi:10.3389/fpsyt.2022.756600)
Supplement: Supplementary file 1 [file Table_1.DOCX]

Supplementary Material

Table A

Gait analysis variables analysed with PODOSmart ®.

|  | MPS | PS | t-test |
| --- | --- | --- | --- |
| right.angle_attack.avg | 0.32 (±0.11) | 0.3 (±0.1) | 0.5166 |
| right. cadence.avg | 45.83 (±4.5) | 46.46 (±4.6) | 0.6596 |
| right. contact_time.avg | 826.53 (±96.64) | 794.19 (±90.03) | 0.2741 |
| right. flight_time.avg | 495.43 (±43.35) | 507.92 (±56) | 0.431 |
| right. oscillation_speed.avg | 1.46 (±0.18) | 1.42 (±0.23) | 0.5184 |
| right. pro_sup_FFI.avg | 0(±0.13) | - 0.05(±0.09) | 0.1446 |
| **right. pro_sup_HO.avg** | **0.06 (±0.11)** | **0 (±0.08)** | **0.0431** |
| right. pro_sup_TO.avg | 0.06 (±0.12) | 0.02 (±0.06) | 0.2032 |
| right. propulsion_speed.avg | 1.13 (±0.24) | 1 (±0.29) | 0.1224 |
| right. stride_length.avg | 1.18 (±0.17) | 1,12 (0.2) | 0.2619 |
| right. stride_length.std | 0.09 (±0.03) | 0.07 (±0.02) | 0.088 |
| right. time_digitigrade_phase.avg | 278.76 (±50.73) | 251.03 (±53.02) | 0.0953 |
| right. walking_speed.avg | 3.25 (±0.54) | 3.11 (±0.63) | 0.4655 |
| right.distance | 42.1 (±13.91) | 39.57 (±16.73) | 0.6025 |
| right.step_count | 35.95 (±9.33) | 39.29 (± 12.57) | 0.8493 |
| left. angle_attack.avg | 0.35 (±0.11) | 0.32 ±(0.11) | 0.4363 |
| left. cadence.avg | 45.91 (±4.01) | 46.7 (±4.54) | 0.5623 |
| left. circumduction.std | 0.02 (±0.01) | 0.02 (±0.01) | 0.7011 |
| left. clearance.avg | 16.23 (±10.42) | 15.86 (±11.42) | 0.9138 |
| left. clearance.std | 5.1 (±1.78) | 5.19 (±1.61) | 0.8629 |
| left. contact_time.avg | 817.77 (±81.45) | 791 (±89.33) | 0.326 |
| left. flight_time.avg | 496.85 (±43.76) | 502.17 (51.66) | 0.7245 |
| left. foot_progression_angle.avg | 0.04 (±0.23) | 0.09 (±0.13) | 0.3562 |
| left. oscillation_speed.avg | 1.49 (±0.15) | 1.4 (±0.22) | 0.1841 |
| left. oscillation_speed.std | 0.11 (±0.04) | 0.09 (±0.03) | 0.2547 |
| left. pro_sup_HO.avg | 0.05 (±0.1) | 0.02 (±0.08) | 0.2536 |
| left. pro_sup_HO.std | 0.02 (±0.01) | 0.03 (±0.01) | 0.4112 |
| left. propulsion_speed.avg | 1.12 (±0.22) | 1.03 (±0.31) | 0.2682 |
| left. propulsion_speed.std | 0.14 (±0.05) | 0.13 (±0.06) | 0.4397 |
| left. stride_length.avg | 1.17 (±0.16) | 1.1 (±0.19) | 0.1927 |
| left. stride_length.std | 0.08 (±0.02) | 0.08 (±0.03) | 0.8275 |
| left. time_digitigrade_phase.avg | 266.46 (±45.57) | 258.01 (±55.71) | 0.5992 |
| left. time_digitigrade_phase.std | 23.09 (±9.4) | 28.47 (±11.33) | 0.1068 |
| left. walking_speed.avg | 3.22 (±0.5) | 3.08 (±0.63) | 0.4109 |
| left. walking_speed.std | 0.32 (±0.1) | 0.29 (±0.1) | 0.4934 |
| left.distance | 41.42 (±13.48) | 38.72 (±16.57) | 0.5719 |
| left.step_count | 36 (±9.86) | 35.48 (±12.84) | 0.8847 |
| right.angle_attack.std * | 0.05 (0.06) | 0.04 (0.05) | 0.1001 |
| right.angle_attack.avg * | 1.79 (4.2) | 1.72 (3.65) | 0.9897 |
| right. circumduction.std * | 0.02 (0.03) | 0.02 (0.03) | 0.6197 |
| right. circumduction.avg * | 0.03 (0.12) | 0.03 (0.08) | 0.8756 |
| right. clearance.avg * | 15.53 (32.52) | 11.68 (48.21) | 0.6147 |
| right. clearance.std * | 5.2 (7.69) | 6.02 (9.34) | 0.3584 |
| right. contact_time.std * | 39.86 (96.34) | 38.79 (53.1) | 0.3862 |
| right. flight_time.std * | 26.75 (43.87) | 21.78 (49.95) | 0.4614 |
| right. foot_progression_angle.avg * | 0.23 (0.78) | 0.21 (0.55) | 0.8043 |
| right. foot_progression_angle.std * | 0.09 (0.09) | 0.09 (0.5) | 0.328 |
| right. gaitline_HO.avg * | 2.96 (1) | 2.57 (1) | 0.3276 |
| right. gaitline_HO.std * | 0.02 (0.45) | 0.1 (0.47) | 0.2466 |
| right. gaitline_HS.avg * | 1.85 (2.09) | 1.53 (2.17) | 0.4106 |
| right. gaitline_HS.avg * | 1.53 (2.17) | 0.38 (0.92) | 0.4106 |
| right. gaitline_HS.std * | 0.38 (0.92) | 0.28 (0.89) | 0.3527 |
| **right. gaitline_TO.avg *** | **3 (0.25)** | **3 (0)** | **0.0356** |
| **right. gaitline_TO.std *** | **0 (0.54)** | **0 (0)** | **0.0356** |
| right. gaitline_TS.avg * | 2.73 (1) | 2.59 (1.02) | 0.5389 |
| right. oscillation_speed.std * | 0.11 (0.12) | 0.09 (0.15) | 0.2105 |
| right. gaitline_TS.std * | 0.2 (0.48) | 0.16 (0.7) | 0.9581 |
| right. pro_sup_FFI.std * | 0.03 (0.04) | 0.03 (0.12) | 0.7049 |
| right. pro_sup_HO.std * | 0.02 (0.05) | 0.03 (0.05) | 0.6198 |
| right. pro_sup_HS.avg * | - 0,14 (0.69) | - 0.16 (0.51) | 0.4894 |
| right. pro_sup_HS.std * | 0.04 (0.05) | 0.04 (0.07) | 0.9896 |
| right. pro_sup_TO.std * | 0.04 (0.11) | 0.05 (0.08) | 0.8244 |
| right. propulsion_speed.std * | 0.15 (0.15) | 0.12 (0.22) | 0.1174 |
| right. time_digitigrade_phase.std * | 22.1 (24.7) | 28.67 (48.48) | 0.2382 |
| right. time_plantigrade_phase.avg * | 396.53 (482.01) | 403.02 (527.24) | 0.5612 |
| right. time_plantigrade_phase.std * | 47.23 (189.26) | 51.71 (183.93) | 0.6891 |
| right. time_taligrade_phase.std * | 11.54 (205.65) | 10.11 (208.9) | 0.3611 |
| right. walking_speed.std * | 0.36 (0.38) | 0.29 (0.39) | 0.1337 |
| right. time_taligrade_phase.avg * | 109.31 (409.39) | 93.03 (421.15) | 1 |
| left. angle_attack.std * | 0.05 (0.05) | 0.04 (0.06) | 0.4413 |
| left. cadence.std * | 1.64 (3.76) | 1.76 (3.49) | 0.6891 |
| left. circumduction.avg * | 0.03 (0.14) | 0.03 (0.1) | 0.8962 |
| left. contact_time.std * | 36.3 (81.74) | 35.81 (62) | 0.5439 |
| left. flight_time.std * | 21.18 (40.03) | 21.98 (35.32) | 0.5612 |
| left. flight_time.std * | 0.07 (35.32) | 0.07 (0.25) | 0.5612 |
| left. foot_progression_angle.std * | 0.07 (0.25) | 0.08 (0.37) | 0.4975 |
| left. gaitline_HO.avg * | 3 (1.01) | 2.93 (0.47 | 0.0787 |
| **left. gaitline_HO.std *** | **0 (0.41)** | **0.15 (0.49)** | **0.0126** |
| left. gaitline_HS.avg * | 1.89 (2.12) | 1.91 (2) | 0.6665 |
| left. gaitline_HS.std * | 0.4 (1) | 0.51 (0.99) | 0.6193 |
| left. gaitline_TO.avg * | 3 (0.18) | 3 (0.24) | 0.7169 |
| left. gaitline_TO.std * | 0 (0.44) | 0 (0.48) | 0.8561 |
| left. gaitline_TS.avg * | 2.81 (1) | 2.71 (1) | 0.627 |
| left. gaitline_TS.std * | 0.1 (0.47) | 0.26 (0.48) | 0.3298 |
| left. pro_sup_FFI.avg * | - O.O1 (0.41) | - 0.02 (0.36) | 0.2848 |
| left. pro_sup_FFI.std * | 0.03 (0.06) | 0.02 (0.11) | 0.9688 |
| left. pro_sup_HS.avg * | - 0.11 (0.66) | - 0.13 (0.48) | 0.5399 |
| left. pro_sup_HS.std * | 0.04 (0.05) | 0.04 (0.05) | 0.8858 |
| left. pro_sup_TO.avg * | - 0.02 (0.47) | - 0.03 (0.22) | 0.3611 |
| left. pro_sup_TO.std * | 0.05 (0.09) | 0.06 (0.08) | 0.784 |
| left. time_plantigrade_phase.avg * | 431.1 (375.21) | 360.46 (524.04) | 0.175 |
| left. time_plantigrade_phase.std * | 46.38 (206.22) | 55.44 (193.7) | 0.9897 |
| left. time_taligrade_phase.avg * | 88.23 (256.45) | 91.35 (425) | 0.3894 |
| left. time_taligrade_phase.std * | 12.97 (207.38) | 12.1 (213.7) | 0.686 |

Variables that presented statistically significant difference (p<0.05) among the MPS and PS groups are indicated with bold letters

Values are Mean (±Standard Deviation) (t-test) or Median (Interquartile Range) in non-normally distributed variables indicated by * (Wilcoxon test)
